# New and existing combinations in Palaeotropical *Phlegmariurus* (Lycopodiaceae) and lectotypification of the type species * Phlegmariurus phlegmaria* (L.) T.Sen & U.Sen.

**DOI:** 10.3897/phytokeys.20.4007

**Published:** 2013-02-26

**Authors:** Ashley R. Field, Peter D. Bostock

**Affiliations:** 1 Australian Tropical Herbarium, James Cook University, Smithfield, Q 4878, Australia; 2 Queensland Herbarium, Department of Science, Information Technology, Innovation and the Arts, Brisbane Botanic Gardens Mt Coot-tha, Toowong Q 4066, Australia

**Keywords:** Lycopodiaceae, Huperzia, Phlegmariurus, Lycopodium, Lycopodiophyta

## Abstract

The genus *Phlegmariurus* Holubis recognised more widely than originally proposed and is circumscribed to include both Neotropic and Palaeotropic epiphytic and terrestrial species of Huperzioid Lycopodiaceae that have isotomous shoots, lack bulbils in their sporophyllous shoots and have spores with convex lateral margins and foveolate-fossulate sculpture restricted to their distal surfaces. New combinations with *Phlegmariurus* are proposed for 81 species and existing combinations identified for 33 species originating from the Palaeotropics. This installs a generic circumscription that is consistent between the Neotropics and Palaeotropics. A lectotype is designated for the type species of the genus, *Phlegmariurus phlegmaria* (L.) T.Sen & U.Sen

## Introduction

The Lycopodiaceae is a globally distributed family of homosporous Lycopodiophytes in which the species exhibit shoot-forms associated with their habitat. Early classifications of this family usually included all of the species, irrespective of their shoot-form, together in the large and polymorphic genus *Lycopodium* L. One exception was the small and morphologically divergent Australian and New Zealand pygmy-clubmoss *Phylloglossum drummondii* Kunzewhich was historically placed in a genus of its own. Multiple genera and subfamilies corresponding with the different shoot-forms, gametophyte and spore types were recognised following multi-character systematic studies on the family (Bruce 1976, Holub 1964, Øllgaard 1975, 1979, 1987, 1989a, Wagner and Beitel 1992, Wilce 1972). Until recently, the most widely adopted of these classifications was the four-genus classification of Øllgaard (1987) which recognised *Lycopodium* L.,* Lycopodiella* Holub,* Huperzia* Bernh.and *Phylloglossum* Kunze and was supported by a comprehensive nomenclatural index (Øllgaard 1989a).

Recent molecular phylogenetic investigations of the family and new morphological observations present a hypothesis that the relationship between *Phylloglossum drummondii* and *Huperzia* is equivocal and that the genus *Huperzia sensu* Øllgaard (1987) is paraphyletic or at best weakly supported(Christenhuszet al. 2011, Jiet al. 2008, Whittier 2006, Whittier and Braggins 1992, 2000, Whittier and Storchova 2007, Wikström and Kenrick 1997, Wikström and Kenrick 2000, 2001, Wikström et al. 1999, Yatsentyuk et al. 2001). A monophyletic *Huperzia* Bernh. is obtained if *Phylloglossum drummondii* is included within it *sensu* Christenhusz et al. (2011). This classification produces a *Huperzia* *sens. lat*. that is morphologically heterogeneous and is not supported by synapomorphies that can be readily identified in the field. It also does not reflect the ancient divergence times between the Huperzioid clades of Lycopodiaceae (Wikström and Kenrick 2001).

An alternative classification is to circumscribe *Huperzia* Bernh. narrowly *sensu* Wagner and Beitel (1992) to include only the wholly terrestrial species with erect shoots bearing gemmae in sporophyll axils and spores that are triangular in polar view with concave lateral margins, truncate corners and foveolate-fossulate sculpture on both proximal and distal surfaces and to recognise a sister genus, *Phlegmariurus* Holub, that is broadened to include all Palaeotropical and Neotropical species with erect or pendulous sporophyllous shoots that lack gemmae and that have spores with convex lateral margins and foveolate-fossulate sculpture restricted to their distal surface. This circumscription of *Phlegmariurus* includes a broad spectrum of both epiphytic and non-epiphytic species primarily from the tropical and subtropical regions globally. The necessary generic combinations with *Phlegmariurus* Holub were recently proposed for the Neotropical species (Øllgaard 2012a, b) but many necessary combinations do not exist among the Palaeotropical species. We propose these transfers so that *Phlegmariurus* Holub may be considered monophyletic.

Species exhibiting the characteristic features of *Phlegmariurus* were identified from the examination of 121 type specimens (including images) for species previously combined with *Lycopodium* L., *Huperzia* Holub and *Urostachys* Herter and from characters described in these species protologues. Species level relationships within Palaeotropical *Phlegmariurus* are poorly understood and their circumscriptions require a thorough molecular and morphological review. It is apparent that there are more species names than there are species, and following a review of type materials we propose a number of new synonyms for species we have studied closely. Molecular evidence suggests that form homoplasy is common, especially among tropical rainforest epiphytes (Wikström et al. 1999). This suggests that morphologically similar species may not always be closely related. We have taken a more conservative approach to synonymisation for the *Phlegmariurus phlegmaria* (L.) T.Sen & U.Senspecies complex as we consider this to be a complex that is rich in species rather than being a remarkably heterogeneous species.

## Lecotypification of *Phlegmariurus phlegmaria* (L.) T.Sen & U.Sen

Epiphytic Lycopodiaceaewere first discovered byEuropean botanists in the East Indies in the late 1600s. Several species were described and illustrated in:

Breyne (1678) *Exoticarum Plantarum Centuria Prima* (tab. 92 *n.v*.)

Rheede et al.(1703) *Hortus Indicus Malabaricus* XII (p. 27, tab. 14!)

Rumph (1741) *Herbarium Amboinense* (VI p. 91, tab. 41, fig. 1!)

Dillen (1741) *Historia Muscorum* (p. 450 tab. 61 fig 5, A, B & C.!)

Dillen (1741) *Historia Muscorum* (p. 450 tab. 61 fig 5, D & E.!)

Linné (1743) *Flora Zeylanica* number 386 (P. Hermann herb. vol. 4. p. 5!)

Linné described *Lycopodium phlegmaria* in *Species Plantarum* in 1753 and cited Dillen (1741 p. 450 tab. 61 fig 5), Linné (1743 p. 183 no. 386 & P. Hermann specimen vol. 4 p. 5), Breyne (1678 tab. 92), and Rheede et al.(1703 p. 27, tab. 14) in his protologue. Based on the limited material available, Linné obviously regarded all of these illustrations, and the P. Hermann specimen, as belonging to the same species. His brief description could be applied to almost any heterophyllous epiphytic species in the genus *Phlegmariurus*. Subsequently, the figures and P. Hermann specimen cited by Linné have been recognised as representing multiple species. In accordance with ICBN (2006) Div. 2, Chapt. 2, Sect. 2 Art. 9.9 a lectotype may be selected to restrict the type of the taxon *Lycopodium phlegmaria* L.to one species (McNeill et al. 2006).

Øllgaard (1989) indicated that Dillen tab. 61 fig 5 A, B & C was eligible for selection as lectotype but did not designate it. The illustrations of Dillen tab. 61 fig 5 ABCDE are based on two separate illustrations reproduced from earlier publications. Dillen tab. 61 fig. 5. A, B & C is reproduced from Rheede et al. (1703 XII p. 27, tab. 14) but Dillen tab. 61 fig. 5. D & E is reproduced from Breyne (1678 tab. 92). In 1810, Willdenow described *Lycopodium mirabilis* (Willd.) Holub for Dillen tab. 61 fig 5 D & E (i.e. the Breyne illustration) but retained the referenceto Dillen tab. 61 fig 5 A, B & C (i.e. the Rheede et al. illustration) for *Lycopodium phlegmaria* L. even though the epithet *phlegmaria* was introduced by Breyne (1678). In 1832, *Lycopodium phyllanthum* Hook. & Arn. was describedfrom new material (Pacific origin) which is in our opinion conspecific with the P. Hermann specimen (Indo-Asian origin). In the interest of conserving nomenclatural stability we believe that Dillen fig. 5, A, B & C. (1741) is the most suitable lectotype because it exhibits characters present in the diagnosis (in accordance with ICBN 2006 Div. 2, Chap. 2, Sect. 2, Art. 9.12.) and most closely accords with *Lycopodium phlegmaria* as it is regarded today (in accordance with ICBN 2006 Div. 2, Chapt. 2, Sect. 2 Art. 9 Recommendation 9.A.5.y) (McNeill et al. 2006).

## Taxonomy

*Phlegmariurus afromontanus* (Pic.Serm.) A.R.Field & Bostock **comb. nov.** Basionym: *Huperzia afromontana* Pic.Serm., Webbia27:394. 1972. Type: Burundi. *J.Lewalle 3468* (holo: FI! [FI3002]). IPNI ID: 77125024-1

*Phlegmariurus aloifolius* (Hook. & Grev.) A.R.Field & Bostock **comb. nov.** Basionym: *Lycopodium aloifolium* Hook. & Grev., Icon. Filic.2:233. 1831. Synonym: *Huperzia aloifolia* (Hook. & Grev.) Trevis., Atti Soc. Ital. Sci. Nat. 17: 248. 1874. Type: India. *N.Wallich 129* (holo: K). IPNI ID: 77125026-1

*Phlegmariurus balansae* (Herter) A.R.Field & Bostock **comb. nov.** Basionym: *Lycopodium balansae* Herter, Bot. Jahrb. Syst. 43: Beibl. 98:51. 1909. Synonym: *Huperzia balansae* (Herter) Holub, Geobot. Phytotax. 20(1): 71. 1985. Type: New Caledonia *M.Balansa 688* (syn: P! [P523007, P523008, P523009]); New Caledonia. *Pompéry s.n.* (syn: P! [P523010]). IPNI ID: 77125028-1

*Phlegmariurus bampsianus* (Pic.Serm.) A.R.Field & Bostock **comb. nov.** Basionym: *Huperzia bampsiana* Pic.Serm., Bull. Jard. Bot. Natl. Belg. 55(1-2): 193. 1985. Type: Rwanda. *J.P.Bamps 2964* (holo: LG; iso: P! [P462093], FI! [FI3007]). IPNI ID: 77125029-1

*Phlegmariurus banayanicus* (Herter) A.R.Field & Bostock **comb. nov.** Basionym: *Urostachys banayanicus* Herter, Bot. Arch. 3:17. 1923. Synonym: *Huperzia banayanica* (Herter) Holub, Geobot. Phytotax. 20(1): 71. 1985.Type: Philippines. *H.M.Curran 8003* (syn: P! [P5239029]); Philippines. *E.B.Copeland 1450* (syn: P! [P523926]); Philippines. *A.D.Elmer 9498* (syn: P! [P523019]); Philippines. *F.W.Foxworthy 2449* (syn: P! [P523018]). IPNI ID: 77125031-1

*Phlegmariurus bolanicus* (Rosenst.) A.R.Field & Bostock **comb. nov.** Basionym: *Lycopodium bolanicum* Rosenst., Repert. Spec. Nov. Regni Veg. 12:181 1913. Synonym: *Huperzia bolanica* (Rosenst.) Holub, Geobot. Phytotax. 20(1): 71. 1985. Type: Papua New Guinea. *C.Keysser B32*. Heterotypic synonyms: *Urostachys kaysseri* Herter *ex* Nessel, Revista Sudamer. Bot. 6:156. 1940; *Huperzia kaysseri* (Herter *ex* Nessel) Holub Folia Geobot. Phytotax. 20(1): 74. 1985. Type: New Guinea. *C.Keysser s.n*. (holo: B [B112944]). IPNI ID: 77125033-1

*Phlegmariurus brachystachys* (Baker) A.R.Field & Bostock **comb. nov.** Basionym: *Lycopodium dacrydioides* Baker. var. *brachystachys* Baker, Handb. Fern-Allies 18. 1887. Synonym: *Huperzia brachystachys* (Baker) Pic.Serm., Webbia 23: 162. 1968. Type: Cameroon. *G.Mann 2041*(holo: K!). IPNI ID: 77125034-1

*Phlegmariurus brassii* (Copel.) A.R.Field & Bostock **comb. nov.** Basionym: *Lycopodium brassii* Copel., J. Arnold Arbor. 10:174 1929. Synonym: *Huperzia brassii* (Copel.) Holub, Geobot. Phytotax. 20(1): 71. 1985. Type: Papua New Guinea. *L.J.Brass 1521* (holo: UC! [UC377989], iso: BRI! [AQ521250]). IPNI ID: 77125036-1

*Phlegmariurus cancellatus* (Spring) Ching, Acta Bot. Yunn. 4:122.1982. Basionym: *Lycopodium cancellatum* Spring, Mém. Acad. Roy. Sci. Belg. 24: 27. 1849. Type: Bhutan. *W.Griffith 162* (holo: K).

*Phlegmariurus carinatus* (Desv. *ex* Poir.) Ching, Acta Bot. Yunn.4:120. 1982.Basionym: *Lycopodium carinatum* Desv. *ex* Poir., Encycl. suppl. 3:555 1813. Synonyms: *Huperzia carinata* (Desv. *ex* Poir.) Trevis., Atti Soc. Ital. Sci. Nat. 17(2) 1874. Type: Sri Lanka. *Jussieu 639* (holo: P!, iso: B!). Heterotypic synonyms: *Lycopodium acrostachyum* Hook. & Grev., Icon Filic. 2:t 181 (1831). Type: Singapore, *N.Wallich 117* (holo: P!). *Lycopodium albescens* Bailey, Queensl. Dept. Agric. Brisbane Bot. Bull. 14:16. 1896; *Huperzia albescens* (F.M.Bailey) Holub, Geobot. Phytotax. 20(1): 70. 1985. Type: Australia ex New Guinea. *M.Sutter ex Capt. Michael s.n*. (holo: BRI! [AQ258306], iso: K). *Lycopodium laxum* C.Presl, Reliq. Haenk. 1:83. 1825; *Huperzia laxa* (C.Presl) Trevis., Atti Soc. Ital. Sci. Nat. 17: 247. 1874; *Phlegmariurus laxus* (C.Presl.) Satou, Hikobia 12(3):268. 1997. Type: Philippines. *Haenke s.n*. (iso: K). *Lycopodium pendulum* Roxb.; Calcutta J. Nat. Hist. 4. 472 (1844). Type: *C. Smith s.n*. from Amboina (lecto: BR *fide* Morton (1974) Contr. U.S. Natl. Herb. 38: 283-396.). *Lycopodium struthioloides* C.Presl, Reliq. Haenk. 1: 82. 1830; *Huperzia struthioloides* (C.Presl) Rothm., Feddes Repert. Spec. Nov. Regni Veg. 54: 61. 1944. Type: Philippines. *Haenke s.n*. (holo: PRC). *Lycopodium vanikorense* Copel., J. Arnold. Arbor. 12:49. 1931; *Huperzia vanikorensis* (Copel.) Holub, Folia Geobot. Phytotax. 20:77. 1985. Type: Vanikoro [Vanuatu]. *S.F.Kajewski 521* (holo: UC! [UC422661], iso: GH, K, MICH, MO! [MO22133], US). 

*Phlegmariurus cavifolius* (C.Chr.) A.R.Field & Bostock **comb. nov.** Basionym: *Lycopodium cavifolium* C.Chr., Dansk Bot. Ark. 7:190. 1932. Synonym: *Huperzia cavifolia* (C.Chr.) Tardieu, Adansonia sér. 2, 10: 18. 1970. Type: Madagascar. *H.Perrier 8273* (holo: BM, iso: P! [P46639], K! [K351220]). *Urostachys smithianus* Nessel, Repert. Spec. Nov. Regni Veg. 36:185. 1934; *Huperzia smithiana* (Nessel) Holub, Geobot. Phytotax. 20(1): 76. 1985. Type: Mauritius: *s.c. s.n*. (syn: BONN-Nessel). IPNI ID: 77125038-1

*Phlegmariurus coralium* (Spring) A.R.Field & Bostock **comb. nov.** Basionym: *Lycopodium coralium* Spring in Miquel, Pl. Jungh. 3: 273. 1854. Synonym: *Huperzia coralium* (Spring) Holub, Folia Geobot. Phytotax. 20(1): 71. 1985. Type: Java [Indonesia]. *Junghuhn 291* (holo: LG, iso: P). IPNI ID: 77125040-1

*Phlegmariurus creber* (Alderw.) A.R.Field & Bostock **comb. nov.** Basionym: *Lycopodium crebrum* Alderw., Bull. Jard. Bot. Buitenzorg ser. 2, 24:4. 1917. Synonym: *Huperzia crebra* (Alderw.) Holub, Geobot. Phytotax. 20(1) 71. 1985. Type: New Guinea. *A.A.Pulle 907* (syn: BM, BO, BONN, K, L, U). IPNI ID: 77125042-1

*Phlegmariurus cryptomerinus* (Maxim.) Satou Hikobia 12 (3): 268. 1997 Basionym: *Lycopodium cryptomerinum* Maxim., Bull. Acad. Sci. St.Petersb. 15: 231. 1870. Synonym: *Huperzia cryptomerina* (Maxim) R.D.Dixit, J. Bombay Nat. Hist. Soc. 77(3): 541. 1980 [1981]. Type: Japan. *Maximowicz s.n*.

*Phlegmariurus cunninghamioides* (Hayata) Ching, Acta Bot. Yunnan.4(2): 120. 1982. Basionym: *Lycopodium cunninghamioides* Hayata, Ic. pl. formos. 4: 131. 1914. Synonym: *Huperzia cunninghamioides* (Hayata) Holub, Geobot. Phytotax. 20(1): 72. 1985. Type: Taiwan. *T.Soma 1* (holo: TI).

*Phlegmariurus curiosus* (Herter) A.R.Field & Bostock **comb. nov.** Basionym: *Urostachys curiosus* Herter, Notul. Syst. 15:355. 1958. Synonym: *Huperzia curiosa* (Herter) Tardieu, Adansonia sér. 2, 10: 18. 1970. Type: Madagascar. *H.Humbert 23478* (holo: P! [P46637]). IPNI ID: 77125044-1

*Phlegmariurus dacrydioides* (Baker) A.R.Field & Bostock **comb. nov.** Basionym: *Lycopodium dacrydioides* Baker, Handb. fern-allies 17-18. 1887. Synonyms: *Huperzia dacrydioides* (Baker) Pic.Serm., Webbia 23: 162. 1968. 1968. Type: Natal. *J.Buchanan s.n*. (lecto: K! [K351207] designated by Pichi Sermolli 1986: 7). IPNI ID: 77125045-1

*Phlegmariurus dalhousieanus* (Spring) A.R.Field & Bostock **comb. nov.** Basionym: *Lycopodium dalhousieanum* Spring, Mem. Acad. Roy. Belg. 24: 25. 1849 Synonym: *Huperzia dalhousieana* (Spring) Trevis., Atti Soc. Ital. Sci. Nat. 17(2) 1874. Type: Penang [Malaysia]. *Lady Dalhousie 36* (holo: K! [3960/59]). Heterotypic synonyms: Based on a morphological comparison of living and herbarium specimens from Indonesia, Malaysia, Papua New Guinea, Australia and Fiji we consider the following taxa to belong to the same widespread but extremely scarce species: *Lycopodium caudifolium* Alderw., Bull. Jard. Bot. Buitenzorg, ser. 2, 1:14. 1911; *Urostachys caudifolius* (Alderw.) Herter *ex* Nessel, Bärlappgewächse 185. 1939; *Huperzia caudifolia* (Alderw.) Holub, Folia Geobot. Phytotax. 20(1): 71. 1985. Type: Borneo [Malaysia]. *A.W.Niewenhuis 317* (holo: BO! [BO185337], iso: L). *Lycopodium clarae* Bailey, Queensl. Dept. Agric. Brisbane Bot. Bull. 7. 1893; *Urostachys clarae* (F.M.Bailey) Herter *ex* Nessel, Bärlappgewächse 185. 1939; *Huperzia clarae* (F.M.Bailey) Holub, Geobot. Phytotax. 20(1): 71. 1985. Type: Australia. *E.Cowley 29* (holo: BRI! [AQ24829]). *Lycopodium glaucum* Cesati, Ann. Acc. Sc. Fis. et. Mat. Napoli VII, 35 (1876); *Urostachys glaucus* (Cesati) Herter, Revista Sudamer. Bot. 8: 86 (1949); Type: Malaysia. *O.Beccari* (holo: FI! [FI11821]). *Lycopodium magnificum* Brownlie, Nova Hedwigia Beih., 55 (Pterid. Fl. Fiji) 22. 1977; *Huperzia magnifica* (Brownlie) Holub, Folia Geobot. Phytotax. 26(1): 92. 1991. Type: Fiji. *Fiji Dept. Agr. 18070* (holo: CHR! [CHR341159]). *Lycopodium ingens* Gepp *ex* Nessel, Bärlappgewächse 185 (1939) nom. nud.; Type: Sogori, New Guinea. *H.O.Forbes s.n*. (holo?: BM! [BM1038045]). IPNI ID: 77125047-1

*Phlegmariurus delbrueckii* (Herter) A.R.Field & Bostock **comb. nov.** Basionym: *Urostachys delbrueckii* Herter, Bot. Arch. 3:19. 1923. Synonyms: *Huperzia delbrueckii* (Herter) Holub, Geobot. Phytotax. 20(1): 72. 1985. Type: Philippines. *E.A.Mearns & W.J.Hutchinson* 4654 (holo: NY! [NY127344], iso: MICH! [MICH1191095], P! [P01220906, P01220907], US! [US134352]). IPNI ID: 77125049-1

*Phlegmariurus dielsii* (Herter) A.R.Field & Bostock **comb. nov.** Basionym: *Lycopodium dielsii* Herter, Bot. Jahrb. Syst. 54:229. 1916. Synonym: *Huperzia dielsii* (Herter) Holub, Geobot. Phytotax. 20(1): 72. 1985. Type: New Guinea. *Ledermann 11318* (syn: B, BM); *R.Schlechter 19085* (syn: B! [B2112382] BONN-Nessel). IPNI ID: 77125051-1

*Phlegmariurus durus* (Pic.Serm.) A.R.Field & Bostock **comb. nov.** Basionym: *Huperzia dura* Pic.Serm., Webbia 27: 392. 1973. Type: Kenya. *R.B.Faden* et al. *70/180* (holo: FI! [FI3008], iso: EA! [EA2516, EA2517], iso: K! [K351206]). IPNI ID: 77125052-1

*Phlegmariurus ellenbeckii* (Nessel) A.R.Field & Bostock **comb. nov.** Basionym: *Urostachys ellenbeckii* Nessel, Repert. Spec. Nov. Regni Veg. 36:188, t. 175. 1934. Synonym: *Huperzia ellenbeckii* (Nessel) Pic.Serm., Webbia 23: 162. 1968. Type: Ethiopia. *Ellenbeck s.n*. (lecto: BONN-Nessel 544 p.p. designated by Pichi Sermolli 1968: 162). IPNI ID: 77125054-1

*Phlegmariurus elmeri* (Herter) A.R.Field & Bostock **comb. nov.** Basionym: *Urostachys elmeri* Herter, Bot. Arch. 3:16. 1923. Synonym: *Huperzia elmeri* (Herter) Holub, Geobot. Phytotax. 20(1): 72. 1985. Type: Philippines. *A.D.Elmer 6411* (isosyn: P! [P01220901]); *J.Bermejos 372* (isosyn: P! [P01220903]); *H.M.Curran 3906* (isosyn: P! [P01220905]); *E.D.Merrill 5923* (isosyn: P! [P01220902]). IPNI ID: 77125056-1

*Phlegmariurus fargesii* (Herter) Ching, Acta Bot. Yunn. 4:125. 1982. Basionym: *Lycopodium fargesii* Herter, Bot. Jahrb. Syst. 43: Beibl. 98: 48. 1909. Synonyms: *Huperzia fargesii* (Herter) Holub, Folia Geobot. Phytotax. 20(1): 72. 1985; *Urostachys fargesii* (Herter) Herter *ex* Nessel, Bärlappgewächse 137. 1939. Type: China. *P.G.Farges s.n*. (holo: P! [P01234387], iso: MO! [MO255690]).

*Phlegmariurus filiformis* (Sw.) W.H.Wagner, Contr. Univ. Michigan Herb.20:242. 1995 Basionym: *Lycopodium filiforme* Sw., J. Bot. (Schrader) 2: 114. 1800 [1801]; Synonyms: *Urostachys filiformis* (Sw.) Herter, Index. Lyc. 61. 1949; *Huperzia filiformis* (Sw.) Holub, Folia Geobot. Phytotax 20:72 (1985). Type: “Insulae Sandwich oceani pacific" [Tahiti] in Syn. Fil. 1806. Heterotypic synonyms: *Lycopodium setaceum* Lam., Encycl. (Lamarck) 3(2): 653. 1792. *Huperzia setacea* (Hamilt. *ex* D. Don) Trevis., Atti Soc. Ital. Sci. Nat. 17:248. 1874. Type: Réunion. *Commerson s.n*. (holo: P! [P46612], iso: P! [P583966]). *Lycopodium polytrichoides* Kaulf., Enum. Filic. 6. 1824; *Huperzia polytrichoides* (Kaulf.) Trevis., Atti Soc. Ital. Sci. Nat. 17(2) 1874. Type: Sandwich Islands [Hawaii], *Chamisso s.n*. (lecto: B designated by Nessel 1939: 30). *Lycopodium verticillatum* L.f.,. Suppl. pl. 448. 1782; *Huperzia verticillata* (L.f.) Rothm., Feddes Repert. Spec. Nov. Regni Veg. 54: 60. 1944. Type: Réunion. *P.Sonnerat per A.Thouin* (holo: SBT! [SBT12294]).

*Phlegmariurus flagellaceus* (Kuhn) A.R.Field & Bostock **comb. nov.** Basionym: *Lycopodium flagellaceum* Kuhn, Forschungsr. Gazelle 4:15. 1889. Synonym: *Huperzia flagellacea* (Kuhn) Holub, Geobot. Phytotax. 20(1): 72. 1985. Type: New Guinea. *Nauman s.n*. (holo: B! [B112446], iso: BM). Note: Some authors have considered this taxon to share affinities with *Phlegmariurus varius* but we consider that it is closer to *Phlegmariurus brassii* (Copel.) A.R.Field & Bostock and the relationship between the two needs investigating. IPNI ID: 77125058-1

*Phlegmariurus foliosus* (Copel.) A.R.Field & Bostock **comb. nov.** Basionym: *Lycopodium foliosum* Copel., Bernice P. Bishop Mus. Bull. 59:7, t.1. 1929. Synonym: *Huperzia foliosa* (Copel.) Holub, Geobot. Phytotax. 20(1): 72. 1985. Type: *H.E.Parks 20651*(holo: UC! [UC351029], iso: BISH! [BISH1000366]). IPNI ID: 77125060-1

*Phlegmariurus fordii* (Baker) Ching, Acta Bot. Yunn.4:126. 1982. Basionym: *Lycopodium fordii* Baker, Handb. Fern-Allies 17. 1887. Synonym: *Huperzia fordii* (Baker) R.D.Dixit Census Indian Pteridophytes 7. 1984. Type: China. *Ford 4* (holo: K).

*Phlegmariurus gagnepainianus* (Herter) A.R.Field & Bostock **comb. nov.** Basionym: *Urostachys gagnepainianus* Herter, Revista Sudamer. Bot. 8:23. 1949. Synonym: *Huperzia gagnepainiana* (Herter) Tardieu, Adansonia sér. 2, 10: 18. 1970. Type: Madagascar. *H.Perrier 15405* (lecto: P! [P226738] designated by Tardieu (1971: 20). IPNI ID: 77125062-1

*Phlegmariurus giganteus* (Herter) A.R.Field & Bostock **comb. nov.** Basionym: *Urostachys giganteus* Herter *ex* Nessel, Bärlappgewächse 124. 1939. Synonym: *Huperzia gigantea* (Herter) Holub, Geobot. Phytotax. 20(1): 73. 1985. Type: Philippines. *E.A.Mearns & Hutchinson 1277* (iso: ?); New Guinea. *MacGregor 3945* (iso: ?). IPNI ID: 77125064-1

*Phlegmariurus gnidioides* (L.f.) A.R.Field & Bostock **comb. nov.** Basionym: *Lycopodium gnidioides* L.f., Suppl. pl. 448. 1782. Synonym: *Huperzia gnidioides* (L.f) Trevis., Atti Soc. Ital. Sci. Nat. 17:247. 1874. Type: Mauritius. *P.Sonnerat per A.Thouin s.n*. (holo: P). Heterotypic synonyms: *Urostachys helmii* Nessel, Repert. Spec. Nov. Regni Veg. 36:186. 1934; *Huperzia helmii* (Nessel) Holub, Geobot. Phytotax. 20(1): 73. 1985. Type: “Insel Greymouth 1885" (lecto: BONN-Nessel designated Øllgaard 1989: 92). IPNI ID: 77125065-1

*Phlegmariurus goebelii* (Nessel) A.R.Field & Bostock **comb. nov.** Basionym: *Urostachys goebelii* Nessel, Repert. Spec. Nov. Regni Veg. 36:188, t.175. 1934. Synonym: *Huperzia goebelii* (Nessel) Holub, Geobot. Phytotax. 20(1): 73. 1985. Type: West-Sumatra [Indonesia] *Goebel s.n*. (isosyn: UC! [UC413367], BONN-Nessel!); Kwala Relai [Malaysia]. *M.Haniff & M.Nur 10225* (isosyn: BM! [BM1038044]). Heterotypic synonyms: *Urostachys heroldii* Herter *ex* Nessel, Revista Sudamer. Bot. 6:167. 1940. Synonym: *Huperzia heroldii* (Nessel) Holub, Geobot. Phytotax. 20(1): 73. 1985. Type: Borneo [Malaysia]. *Hallier* (holo: P?). IPNI ID: 77125067-1

*Phlegmariurus guandongensis* Ching, Acta Bot. Yunnan.4(2):123. 1982. Type: China. *S.P.Ko 51291* (holo: PE).

*Phlegmariurus gunturensis* (Alderw.) A.R.Field & Bostock **comb. nov.** Basionym: *Lycopodium gunturense* Alderw., Bull. Jard. Bot. Buitenzorg ser. 2, 1:14. 1911. Synonym: *Huperzia gunturensis* (Alderw.) Holub, Folia Geobot. Phytotax. 20(1): 73. 1985. Type: Indonesia. *A.E.Kerkhoven 15* (holo: BO [BO1826257]). IPNI ID: 77125069-1

*Phlegmariurus hamiltonii* (Spreng.) Á.Löve & D.Löve, Taxon26(2-3):324. 1977. Basionym: *Lycopodium hamiltonii* Spreng., Syst. veg. 5: 429. 1828. Type: Nepal. *J.Buchanan s.n*. (holo: BM).

*Phlegmariurus harmsii* (Nessel) A.R.Field & Bostock **comb. nov.** Basionym: *Urostachys harmsii* Herter *ex* Nessel, Revista Sudamer. Bot. 6: 166. 1940. Synonym: *Huperzia harmsii* (Herter *ex* Nessel) Holub, Geobot. Phytotax. 20(1): 73. 1985. Type: Caroline Islands [Micronesia] *R.Kanehira 1316* (iso: NY! [NY127353], US [US1917849]). IPNI ID: 77125071-1

*Phlegmariurus hellwigii* (Warb.) A.R.Field & Bostock **comb. nov.** Basionym: *Lycopodium hellwigii* Warb., Monsunia, 1:97. 1898. Synonym: *Huperzia hellwigii* (O.Warb.) Holub, Geobot. Phytotax. 20(1): 73. 1985. Type: Papua New Guinea. *F.Hellwig 349* (holo: B! [B112544], iso: BONN-Nessel). IPNI ID: 77125073-1

*Phlegmariurus henryi* (Baker) Ching, Acta Bot. Yunnan.4(2):125 1982. Basionym: *Lycopodium henryi* Baker, Kew Bull. 1906:15. 1906. Type: China. *Henry 11551* (holo: K).

*Phlegmariurus hillianus* (Nessel) A.R.Field & Bostock **comb. nov.** Basionym: *Urostachys hillianus* Nessel, Repert. Spec. Nov. Regni Veg. 36:16. 1934. Synonym: *Huperzia hilliana* (Nessel) Holub, Geobot. Phytotax. 20(1): 73. 1985. Type: India. *H.C.Levinge s.n*. (holo: K, iso: BONN-Nessel). IPNI ID: 77125075-1

*Phlegmariurus holstii* (Hieron.) A.R.Field & Bostock **comb. nov.** Basionym: *Lycopodium holstii* Hieron. in Engler, Pflanzenw. Ost-Afrikas C:90. 1985. Synonyms: *Huperzia holstii* (Hieron.) Pic.Serm., Webbia 23: 163. 1968. Type: Usambara [Tanzania]. *C.Holst 8814* (holo: B! [B112586], iso: K! [K351205], P! [P466630], BM! [BM785226]). IPNI ID: 77125077-1

*Phlegmariurus horizontalis* (Nessel) A.R.Field & Bostock **comb. nov.** Basionym: *Urostachys horizontalis* Nessel, Bärlappgewächse 225. 1939. Synonym: *Huperzia horizontalis* (Herter *ex* Nessel) Holub, Geobot. Phytotax. 20(1): 73. 1985. Type: Borneo [Malaysia]. *A.W.Niewenhuis 311* (iso: L! [L57351], BONN-Nessel). IPNI ID: 77125079-1

*Phlegmariurus humbertii* (Nessel) A.R.Field & Bostock **comb. nov.** Basionym: *Urostachys humbertii* Nessel, Repert. Sp. Nov. Regni Veg. 48:170. 1940. Type: Madagascar. *J.Kiese s.n*. (holo: BONN-Nessel). IPNI ID: 77125546-1

*Phlegmariurus humbertii-henrici* (Herter) A.R.Field & Bostock **comb. nov.** Basionym: *Urostachys humbertii-henrici* Herter, Revista Sudamer. Bot. 8:23. 1949. Synonym: *Huperzia humbertii-henrici* (Herter) Tardieu, Adansonia sér. 2, 10: 18. 1970. Type: Madagascar. *H.Humbert 12224* (isolecto: P! [P466628, P466627, P466629] designated by Tardieu 1971: 29). IPNI ID: 77125081-1

*Phlegmariurus jaegeri* (Herter) A.R.Field & Bostock **comb. nov.** Basionym: *Urostachys jaegeri* Herter, Revista Sudamer. Bot. 8:24. 1949. Synonym: *Huperzia jaegeri* (Herter) Pic.Serm., Webbia 23: 163. 1968. Type: Sierra Leone. *Jaeger 1522* (holo: P! [P466622]). IPNI ID: 77125083-1

*Phlegmariurus juniperistachyus* (Hayata) A.R.Field & Bostock **comb. nov.** Basionym: *Lycopodium juniperistachyum* Hayata, Ic. Pl. Formos. 4:132. 1914. Synonym: *Huperzia juniperistachya* (Hayata) Holub, Geobot. Phytotax. 20(1): 74. 1985. Type: Taiwan. *Kawakami & Nakahara 787* (holo: TI). IPNI ID: 77125085-1

*Phlegmariurus lauterbachii* (E.Pritz. *ex* Schumann & Lauterb.) A.R.Field & Bostock **comb. nov.** Basionym: *Lycopodium lauterbachii* E.Pritz. *ex* Schumann & Lauterb., Südsee 149. 1900. Synonym: *Huperzia lauterbachii* (E.Pritz.) Holub, Geobot. Phytotax. 20(1): 74. 1985. Type: New Guinea. *C.A.G.Lauterbach 3174* (holo: B, iso: BONN-Nessel). IPNI ID: 77125087-1

*Phlegmariurus lecomteanus* (Nessel) A.R.Field & Bostock **comb. nov.** Basionym: *Urostachys lecomteanus* Nessel, Repert Spec. Nov. Regni Veg. 36(12-15):187. 1934. Synonyms: *Huperzia lecomteana* (Nessel) Holub, Geobot. Phytotax. 20(1): 74. 1985. Type: Madagascar. (holo: BONN-Nessel 492). IPNI ID: 77125089-1

*Phlegmariurus ledermannii* (Herter) A.R.Field & Bostock **comb. nov.** Basionym: *Lycopodium ledermannii* Herter, Bot. Jahrb. Syst. 54:232. 1916. Synonym: *Huperzia ledermannii* (Herter) Holub, Geobot. Phytotax. 20(1): 74. 198. Type: New Guinea. *C.Keysser 199* (iso: SUNIV!, B! [B113004]). IPNI ID: 77125091-1

*Phlegmariurus lockyeri* (D.L.Jones & B.Gray ), A.R.Field & Bostock **comb. nov.** Basionym: *Lycopodium lockyeri* D.L.Jones & B. Gray, Austrobaileya 2:126. 1985. Synonym: *Huperzia lockyeri* (D.L.Jones & B.Gray) Holub, Geobot. Phytotax. 26(1): 92. 1991. Type: Australia *B.Gray 3541* (holo: QRS! [QRS77021]) IPNI ID: 77125092-1

*Phlegmariurus longus* (Copel.), A.R.Field & Bostock **comb. nov.** Basionym: *Lycopodium longum* Copel., Philipp. J. Sci. 60:100, t.3. 1936. Type: Solomon Islands. *S.F.Kajewski 1953* (holo: PHN destroyed, iso: BM! [BM16725], MICH! [MICH1287171]). IPNI ID: 77125094-1

*Phlegmariurus lonyangensis* C.Y.Ma, Bull. Bot. Res., Harbin 10(3):58. 1990. Type: China *C.Y.Ma 3246* (holo: AMMS).

*Phlegmariurus macgregorii* (Baker) A.R.Field & Bostock **comb. nov.** Basionym: *Lycopodium macgregorii* Baker, J. Bot. 28: 109. 1890. Synonym: *Huperzia macgregorii* (Baker) Holub, Geobot. Phytotax. 20(1): 74. 1985. Type: New Guinea. *W.MacGregor 68* (holo: K!, iso: BM! [BM1038027, BM1038028]). Heterotypic synonyms: *Lycopodium versteegii* Alderw., Bull. Jard. Bot. Buitenzorg, ser. 2, 24:4. 1917; *Huperzia versteegii* (Alderw.) Holub, Geobot. Phytotax. 20(1): 77. 1985. Type: (holo: New Guinea. *G.M.Vertseeg 2510* (holo: U! [U7449]). *Urostachys whartoniensis* Nessel, Repert. Spec. Nov. Regni Veg. 38:62. 1935. *Huperzia whartoniensis* (Nessel) Holub, Geobot. Phytotax. 20(1): 78. 1985. Type: Papua New Guinea. *L.J.Brass 4729* (iso: NY! [NY127367], iso: BRI! [AQ24833]). IPNI ID: 77125096-1

*Phlegmariurus macrostachys* (Hook. *ex* Spring) N.C.Nair & S.R.Ghosh, J. Econ. Taxon.Bot.12(1):194. 1988. Synonyms: *Huperzia macrostachys* (Hook. *ex* Spring) Holub, Geobot. Phytotax. 20(1): 74. 1985. Type: India. *F.Adams s.n*. (syn: K). Sri Lanka. *Walker s.n*. (syn: K).

*Phlegmariurus mannii* (Hillebr.) W.H.Wagner, Contr. Univ. Michigan Herb. 20:241 1995. Basionym: *Lycopodium phlegmaria* var. *mannii* Hillebr., Fl. Hawaiian Isl. 645. 1888. Type: Hawaii. *H.Mann & W.T.Brigham 506* (iso: BISH! [BISH1005485], MO! [MO22681]).

*Phlegmariurus marsupiiformis* (D.L.Jones & B.Gray) A.R.Field & Bostock **comb. nov.** Basionym: *Lycopodium marsupiiforme* D.L.Jones & B.Gray, Austrobaileya 2:128. 1985. Synonym: *Huperzia marsupiiformis* (D.L.Jones & B.Gray) Holub, Geobot. Phytotax. 26(1): 92. 1991. Type: Australia. *B.Gray 3841* (holo: BRI! [AQ419317], CNS! [QRS78343]). IPNI ID: 77125098-1

*Phlegmariurus megastachyus* (Baker) A.R.Field & Bostock **comb. nov.** Basionym: *Lycopodium megastachyum* Baker, J. Linn. Soc. Bot. 21:454. 1885. Synonym: *Huperzia megastachya* (Baker) Tardieu, Adansonia sér. 2, 10: 18. 1970. Type: Madagascar. *R.Baron 2840* (holo: K! [K351218, K351219]). IPNI ID: 77125100-1

*Phlegmariurus merrillii* (Herter) A.R.Field & Bostock **comb. nov.** Basionym: *Urostachys merrillii* Herter, Bot. Arch. 3:15. 1923. Synonym: *Huperzia merrillii* (Herter) Holub, Geobot. Phytotax. 20(1): 74. 1985. Type: Philippines. *E.D.Merrill 965* (iso: UC! [UC1096474], U! [U7444], MICH! [MICH1191096]). IPNI ID: 77125103-1

*Phlegmariurus milbraedii* (Herter) A.R.Field & Bostock **comb. nov.** Basionym: *Lycopodium milbraedii* Herter, Hedwigia 49:90. 1909. Synonym: *Huperzia milbraedii* (Herter) Pic.Serm., Webbia 23:163. 1968. Type: Cameroon. *Milbraed 3449* (holo: B). IPNI ID: 77125104-1

*Phlegmariurus mingcheensis* Ching, Acta Bot. Yunnan.4(2): 125. 1982. Synonym: *Huperzia mingcheensis* (Ching) Holub, Folia Geobot. Phytotax. 20: 74. 1985. Type: China. *P.S.Chiou 2069* (holo: PE). See recent note on the nomenclature of this species (Zhang 2012).

*Phlegmariurus minutifolius* (Alderw.) A.R.Field & Bostock **comb. nov.** Basionym: *Lycopodium coralium* var. *minutifolium* Alderw., Malayan fern allies 44. 1915. Synonyms: *Huperzia minutifolia* (Alderw.) Holub, Folia Geobot. Phytotax 20(1): 74. 1985. Type: New Guinea. *Ruchmat s.n*. (holo: L). IPNI ID: 77125106-1

*Phlegmariurus multifarius* (Alderw.) A.R.Field & Bostock **comb. nov.** Basionym: *Lycopodium multifarium* Alderw., Bull. Jard. Bot. Buitenzorg, ser. 3, 5:226. 1922. Synonym: *Huperzia multifaria* (Alderw.) Holub, Geobot. Phytotax. 20(1): 75. 1985. Type: Ternate [Moluccas, Indonesia]. *V.M.A.Beguin 1329* (holo: BO [BO186327]). IPNI ID: 77125108-1

*Phlegmariurus myrtifolius* (G.Forst.) A.R.Field & Bostock **comb. nov.** Basionym: *Lycopodium myrtifolium* G.Forst., Florae Insulae Australiae Prodromus. 1786. Synonym: *Huperzia myrtifolia* (G.Forst.) Holub, Geobot. Phytotax. 20(1): 75. 1985. Type: New Caledonia. *G.Forster s.n*. (iso: P-Luerss., UPS-Thunb). IPNI ID: 77125109-1

*Phlegmariurus nanus* C.Y.Ma, *Bull. Bot. Res., Harbin* 10(3):58. 1990. Type: China. *C.Y.Ma 3274* (holo: AMMS).

*Phlegmariurus neocaledonicus* (Nessel) A.R.Field & Bostock **comb. nov.** Basionym: *Urostachys neocaledonicus* Nessel, Repert. Spec. Nov. Regni Veg. 36:187. 1934. Synonym: *Huperzia neocaledonica* (Nessel) Holub, Geobot. Phytotax. 20(1): 75. 1985. Type: New Caledonia. *E.Viellard* (lecto: BONN-Nessel designated Øllgaard 1989: 96). IPNI ID: 77125110-1

*Phlegmariurus nilagiricus* (Spring) A.R.Field & Bostock **comb. nov.** Basionym: *Lycopodium nilagiricum* Spring, Bull. Acad. Roy. Sci. Belg. 8:517. 1841. Synonym: *Huperzia nilagirica* (Spring) Dixit, J. Bombay Nat. Hist. Soc. 77(3):541. 1981. Type: India. *Perottet* (holo: G-Deless.). IPNI ID: 77125112-1

*Phlegmariurus nummulariifolius* (Blume) Ching, Acta Bot. Yunnan.4(2):125. 1982. Basionym: *Lycopodium nummulariifolium* Blume, Enum. pl. Javae 2: 263. 1828. Synonym: *Huperzia nummulariifolia* (Blume) Chambers, Jermy & Crabbe, Brit. Fern Gaz. 10: 176. 1971. Type: Java. *C.L.Blume s.n*. (isolecto: L! [L57363]).

*Phlegmariurus nutans* (Brack.) W.H.Wagner Contr. Univ. Michigan Herb.20:241. 1995. Synonym: *Huperzia nutans* (Brack.) Rothm., Feddes Repert. 54: 62. 1944. Type: Hawaii. *Wilkes 10* (holo: US, iso: BISH! [BISH1005484]).

*Phlegmariurus nylamensis* (Ching & S.K.Wu) H.S.Kung & Li Bing Zhang, Acta Phytotax. Sin.37(1):52. 1999.

*Phlegmariurus obtusifolius* (P.Beauv.) A.R.Field & Bostock **comb. nov.** Basionym: *Lepidotis obtusifolia* P.Beauv., Prodr. Aethéogam. 109. 1805. Synonym: *Huperzia obtusifolia* (P.Beauv.) Rothm., Feddes Repert. 54:61. 1944. Type: Réunion. *J.B.G.M. Bory de St. Vincent* (holo: P! [P466623], iso: B-Wild.! [B-W19344]). Heterotypic synonyms: *Lycopodium pachyphyllum* Kuhn *ex* Herter, Bot. Jahrb. Syst. 43: Beibl. 98:51. 1909; *Huperzia pachyphylla* (Kuhn) Holub, Geobot. Phytotax. 20(1): 75. 1985. Type: Madagascar. *J.M.Hildebrandt* 4141(holo: B! [B85521], iso: B! [B8522], P! [P466624, P466625, P46626]). IPNI ID: 77125135-1

*Phlegmariurus oceanianus* (Herter) A.R.Field & Bostock **comb. nov.** Basionym: *Lycopodium oceanianum* Herter, Bot. Jahrb. Syst. 43: Beibl. 98:52. 1909. Synonym: *Huperzia oceaniana* (Herter) Holub, Folia Geobot. Phytotax. 20:75.1985. Type: New Hebrides [Vanuatu] *s.c. s.n*. (syn: P). IPNI ID: 77125167-1

*Phlegmariurus oltmannsii* (Herter *ex* Nessel) A.R.Field & Bostock **comb. nov.** Basionym: *Urostachys oltmannsii* Herter *ex* Nessel [Bärlappgewächse 231. 1939, nom. inval.] Revista Sudamer. Bot. 6: 168. 1940. Synonym: *Huperzia oltmannsii* (Herter *ex* Nessel) Holub, Geobot. Phytotax. 20(1): 75. 1985. Type: Tahiti. *J.McGillivray 1331*. IPNI ID: 77125115-1

*Phlegmariurus ophioglossoides* (Lam.) A.R.Field & Bostock **comb. nov.** Basionym: *Lycopodium ophioglossoides* Lam., Encycl. (Lamarck) 3:646. 1789. Synonym: *Huperzia ophioglossoides* (Lam.) Rothm., Feddes Repert. 54:62. 1944. Type: Mauritius. *Commerson s.n*. (holo: P-Lam). Note: Molecular phylogenetic evidence indicates that this Palaeotropic species belongs to a Neotropical radiation of *Phlegmariurus*. IPNI ID: 77125118-1

*Phlegmariurus ovatifolius* (Ching) W.M.Chu *ex* H.S.Kung & Li Bing Zhang, Acta Phytotax. Sin.37(1):52 (1999). Basionym: *Huperzia ovatifolia* Ching, Acta Bot. Yunnanica 3: 298. 1981. Type: China. *Yunnan Complex Exped. 1504* (holo: PE).

*Phlegmariurus parksii* (Copel.) A.R.Field & Bostock **comb. nov.** Basionym: *Lycopodium parksii* Copel., Bernice P. Bishop. Mus. Bull. 59:8, t.2. 1929. Synonym: *Huperzia parksii* (Copel.) Holub, Geobot. Phytotax. 20(1): 75. 1985. Type: Fiji. *H.E.Parks 20584* (holo: UC! [UC351026], iso: P! [P711367], BISH! [BISH1000370], US! [US1759872]). Heterotypic synonym: *Urostachys bonapartei* Nessel, Repert. Spec. Nov. Regni Veg.. 36:182 t.172 1934; *Huperzia bonapartei* (Nessel) Holub, Geobot. Phytotax. 20(1): 71. 1985. Type: Flora Vitiensis [Fiji] *C.Weber s.n*. (syn: BONN). IPNI ID: 77125120-1

*Phlegmariurus patentissimus* (Alderw.) A.R.Field & Bostock **comb. nov.** Basionym: *Lycopodium patentissimum* Alderw., Bull. Jard. Bot. Buitenzorg, ser. 2, 24:4. 1917. Synonym: *Huperzia patentissima* (Alderw.) Holub, Geobot. Phytotax. 20(1): 75. 1985. Type: Irian Jaya [Indonesia]. *A.A.Pulle 462* (holo: BO, iso: BM, BONN-Nessel, L! [L57365], U). IPNI ID: 77125121-1

*Phlegmariurus pecten* (Baker) A.R.Field & Bostock **comb. nov.** Basionym: *Lycopodium pecten* Baker, J. Linn. Soc. Bot. 15:421. 1876. Synonym: *Huperzia pecten* (Baker) Tardieu, Adansonia sér. 2, 10: 18. 1970. Type: Madagascar. *W.Pool s.n*. (holo: K1 [K351214]). IPNI ID: 77125123-1

*Phlegmariurus perrerianus* (Tardieu) A.R.Field & Bostock **comb. nov.** Basionym: *Huperzia perreriana* Tardieu, Adansonia 10:20. 1970. Type: Madagascar. *H.Perrier 8294* (holo: P). IPNI ID: 77125127-1

*Phlegmariurus petiolatus* (C.B.Clarke) H.S.Kung & Li Bing Zhang, Acta Phytotax. Sin.37(1): 52. 1999. Basionym: *Lycopodium hamiltonii* var. *petiolatus* C.B.Clarke, Trans. Linn. Soc. London, ser. 2, Bot. 1: 590. 1880. Synonyms: *Huperzia petiolata* (C.B.Clarke) R.D.Dixit, J. Bombay Nat. Hist. Soc. 77(3): 541. 1980 [1981]. Type: Khasia [Bangladesh]. *C.B.Clarke s.n*. (holo: BM).

*Phlegmariurus phlegmaria* (L.) T.Sen & U.Sen, Fern. Gaz.11(6):421. 1978. Basionym: *Lycopodium phlegmaria* L., Species Plantarum 2. 1753. Synonym: *Huperzia phlegmaria* (L.) Rothm., Feddes Repert. 54: 62. 1944. Type: India. *s.c. s.n*. (lecto: Dillen (1741) *Historia Muscorum* p. 450 tab. 61 fig 5, A, B & C.! **here designated**).

*Phlegmariurus phlegmarioides* (Gaudich.) A.R.Field & Bostock **comb. nov.** Basionym: *Lycopodium phlegmarioides* Gaudich., Voy. Uranie, Bot. 1830. Synonym: *Huperzia phlegmarioides* (Gaudich.) Rothm., Feddes Repert. Spec. Nov. Regni Veg. 54(1). 1944. Type: Moluccas [Indonesia] *C.-B.Gaudichaud s.n*. (holo: P!, iso: K!). Heterotypic synonyms: *Urostachys kandavuensis* Nessel, Repert. Spec. Nov. Regni Veg. 39:67. 1935; *Huperzia kandavuensis* (Nessel) Holub, Geobot. Phytotax. 20(1): 74. 1985. Type: Fiji. *A.C.Smith 142* (holo: NY! [NY127357]). *Lycopodium pseudophlegmaria* Kuhn, Forschungsr. Gazelle 4: 16. 1889; *Urostachys pseudophlegmaria* (Kuhn) Herter *ex* Nessel, Bärlappgewächse 237. 1939; *Huperzia pseudophlegmaria* (Kuhn) Holub, Folia Geobot. Phytotax. 20: 76. 1985; Type: *Naumann*, Fiji Islands (holo - unknown). *Lycopodium tetrapterygium* F.M.Bailey, Proc. Roy. Soc. Queensland 1:150 (1885); *Urostachys tetrapterygius* (F.M.Bailey) Herter, Index Lyc. 86. 1949; Type: Daintree River, Australia *Fitzalan s.n*. (syn: BRI!) and Johnstone River, Australia *Kefford s.n*. (syn: BRI!). IPNI ID: 77125129-1

*Phlegmariurus phyllanthus* R.D.Dixit J. Bombay Nat. Hist. Soc. 77(3): 541. 1981. Synonyms: *Huperzia phyllantha* (Hook. & Arn.) Holub, Geobot. Phytotax. 20(1): 75. 1985. Type: Hawaiian Islands *G.T.Lay & A.Collie 3321* (holo: BM! [BM1038070]). Heterotypic synonyms: *Lycopodium pachystachyon* Spring, Bull. Acad. Roy. Sci. Belg. 8:518. 1841. *Huperzia pachystachyon* (Spring) Roth., Feddes Repert. 54:62. 1944. Type: Hawaii. *C.-B.Gaudichaud* (iso: BM, L! [L57364], P). IPNI ID: 77125132-1

*Phlegmariurus pichianus* (Tardieu) A.R.Field & Bostock **comb. nov.** Basionym: *Huperzia pichiana* Tardieu, Adansonia sér. 2, 10: 21, t. 2, f. 5-8. 1970. Type: Madagascar. *H.Perrier 17773* (holo: P! [P466620], iso: BM! [BM785232], P! [P466618, P466619, P226993]). IPNI ID: 77125134-1

*Phlegmariurus proliferus* (Blume) A.R.Field & Bostock **comb. nov.** Basionym: *Lycopodium proliferum* Blume, Enum. Pl. Javae 2:265. 1828. Synonym: *Huperzia prolifera* (Blume) Trevis., Atti Soc. Ital. Sci. Nat. 17(2). 1874. Type: Java [Indonesia], *C.L.Blume s.n*. (lecto: L! [L57380], iso: P!). IPNI ID: 77125135-1

*Phlegmariurus pulcherrimus* Á.Löve & D.Löve Taxon 26(2-3): 324. Basionym: *Lycopodium pulcherrimum* Wall. *ex* Hook. & Grev. Icon. Filic. 1(2): 38. 1827. Synonyms: *Huperzia pulcherrima* (Wall. *ex* Hook. & Grev.) Pic.Serm. Type: Nepal *N. Wallich s.n*. (iso: BM! [BM1038025]). IPNI ID: 77125138-1

*Phlegmariurus ribourtii* (Herter) A.R.Field & Bostock **comb. nov.** Basionym: *Lycopodium ribourtii* Herter, Bot. Jahrb. Syst. 43: Beibl. 98:53. 1909. Synonym: *Huperzia ribourtii* (Herter) Holub, Geobot. Phytotax. 20(1): 76. 1985. Type: Tahiti. *Ribourt s.n*. (syn: P, BONN-Nessel), *Nadeau s.n*. (syn: P). IPNI ID: 77125140-1

*Phlegmariurus rubricus* (Herter) A.R.Field & Bostock **comb. nov.** Basionym: *Urostachys rubricus* Herter, Revista Sudamer. Bot. 8:25. 1949. Synonym: *Huperzia rubrica* (Herter) Tardieu, Adansonia sér. 2, 10: 18. 1970. Type: Madagascar. *H.Humbert 3717* (holo: P! [P466617]). IPNI ID: 77125141-1

*Phlegmariurus rupicola* (Alderw.) A.R.Field & Bostock **comb. nov.** Basionym: *Lycopodium rupicola* Alderw., Bull. Jard. Bot. Buitenzorg, ser. 2, 16:39. 1914. Synonym: *Huperzia rupicola* (Alderw.) Holub, Geobot. Phytotax. 20(1): 76. 1985. Type: New Guinea. *Gjellerup 1080* (holo: BO, iso: L! [L57385]). IPNI ID: 77125145-1

*Phlegmariurus salvinioides* (Herter) Ching, Acta Bot. Yunnan. 4(2):122. 1982. Basionym: *Urostachys salvinioides* Herter, Bot. Arch. 3: 18. 1923. Synonym: *Huperzia salvinioides* (Herter) Holub, Geobot. Phytotax. 20(1): 76. 1985. Type: not designated, numerous Philippines specimens cited. Heterotypic synonyms: *Lycopodium edanoi* Copel., Philipp. J. Sci. 46:209. 1931. Synonym: *Huperzia edanoi* (Copel.) Holub, Geobot. Phytotax. 20(1): 72. 1985. Type: Philippines *G.E.Edaño* (holo: MICH, iso: NY! [NY127347]).

*Phlegmariurus samoanus* (Herter *ex* Nessel) A.R.Field & Bostock **comb. nov.** Basionym: *Urostachys samoanus* Herter *ex* Nessel, Revista Sudamer. Bot. 6:163. 1940. Synonyms: *Huperzia samoana* (Nessel) Holub, Geobot. Phytotax. 20(1): 76. 1985. Type: Samoa. *Dr Friedrich s.n*. (lecto: BONN-Nessel designated Øllgaard 1989: 100). IPNI ID: 77125146-1

*Phlegmariurus shangsiensis* C.Y.Yang, Acta Phytotax. Sin.22(1):87. 1984. Type: China. *W.T.Tsan 22289* (holo: SCBI).

*Phlegmariurus schlechteri* (E.Pritz.) A.R.Field & Bostock **comb. nov.** Basionym: *Lycopodium schlecteri* E.Pritz. in Schechter, Bot. Jahrb. Syst. 39:14. 1906. Synonym: *Huperzia schlechteri* (E.Pritz.) Holub, Geobot. Phytotax. 20(1): 76. 1985. Type: New Caledonia. *R.Schlechter 15174* (holo: B, iso: BM, K). IPNI ID: 77125148-1

*Phlegmariurus setifolius* (Alderw.) A.R.Field & Bostock **comb. nov.** Basionym: *Lycopodium setifolium* Alderw., Bull. Bot. Jahrb. Buitenzorg, ser. 2, 16:40. 1914. Synonym: *Huperzia setifolia* (Alderw.) Holub, Geobot. Phytotax. 20(1): 76. 1985. Type: Borneo. *Teysman s.n*. (iso: L! [L57387]). IPNI ID: 77125150-1

*Phlegmariurus sieboldii* (Miq.) Ching, Fl. Xisang 1:12. 1983. Basionym: *Lycopodium sieboldii* Miq., Ann. Mus. Bot. Lugduno-Batavum 3: 184: 1897. Synonyms: *Huperzia sieboldii* (Miq.) Holub, Geobot. Phytotax. 20(1): 76. 1985. Type: Japan. *P.F.von Siebold s.n*. (iso: L, US! [US134339]).

*Phlegmariurus sooianus* Lawalrée, Acta Bot. Acad. Sci. Hung. 19: 195. 1973. Synonyms: *Huperzia sooiana* (Lawalrée) Holub, Folia Geobot. Phytotax. 20: 77. 1985. Type: Zaire. *Evrard 3580* (holo: BR).

*Phlegmariurus squarrosus* (G.Forst.) Á. Löve & D. Löve, Taxon 26:324. 1977. Basionym: *Lycopodium squarrosum* G.Forst., Fl. Ins. Austr. 479 (1786). Type: Tahiti. *J & G. Forster s.n*. (isosyn: BM! [BM1048443] and P!). Synonyms: *Huperzia squarrosa* (G.Forst.) Trevis.; Atti Soc. Ital. Sci. Nat. 17: 247. 1874; *Urostachys squarrosus* (G.Forst.) Herter; Bot. Arch. 3: 14. 1923.Heterotypic synonyms: *Lycopodium acutifolium* Desv. *ex* Poir., Encycl. suppl. 3: 599. 1813 [1814]. Type: Mauritius. *s.c. s.n*. (holo: P! [P4666615]). *Lycopodium epiceifolium* Desv. *ex* Poir., Encycl. suppl. 3:559. 1913; *Huperzia epiceifolia* (Desv. *ex* Poir.) Trevis., Atti Soc. Ital. Sci. Nat. 17: 248. 1874. Type: Mauritius. *s.c. s.n*. (holo: P! [P0466614]). *Urostachys madagascariensis* (Nessel) Herter, Index Lyc. 68. 1949; Type: Madagascar. *H.Perrier 8276* (holo: P). *Lycopodium pseudosquarrosum* Pamp., Bull. Reale Soc. Tosc. Ortic. 111, 13: 99. 1908. Type: Indonesia. *Pampanini s.n*. (iso: P).

*Phlegmariurus staudtii* (Nessel) A.R.Field & Bostock **comb. nov.** Basionym: *Urostachys staudtii* Nessel, Repert. Spec. Nov. Regni Veg. 48:170. 1940. Type: Cameroon. *Staudt 476* (lecto: P, isolecto: BONN-Nessel). IPNI ID: 77125151-1

*Phlegmariurus strictus* (Baker) A.R.Field & Bostock **comb. nov.** Basionym: *Lycopodium strictum* Baker, J. Bot. 11:271. 1882. Synonym: *Huperzia stricta* (Baker) Tardieu, Adansonia sér. 2, 10: 20. 1970. Type: Madagascar. *Parker s.n*. (holo: K). IPNI ID: 77125152-1

*Phlegmariurus subfalciformis* (Alderw.) A.R.Field & Bostock **comb. nov**. Basionym: *Lycopodium subfalciforme* Alderw., Bull. Jard. Bot. Buitenzorg. ser. 2, 28:44. 1918. Synonym: *Huperzia subfalciformis* (Alderw.) Holub, Geobot. Phytotax. 20(1): 77. 1985. Type: Papua New Guinea. *R.Schlechter 13775* (syn: B! [B0114953]). IPNI ID: 77125153-1

*Phlegmariurus subulifolius* (Wall. *ex* Hook. & Grev.) S.R.Ghosh, Pterid. Fl. E. India66. 2004. Basionym: *Lycopodium subulifolium* Wall. *ex* Hook. & Grev., Icon. Filic. 1. 1827. Synonym: *Huperzia subulifolia* (Wall. *ex* Hook & Grev.) Trevis., Atti Soc. Ital. Sci. Nat. 17: 248. 1874. Type: Nepal. *N.Wallich s.n*. (holo: K). IPNI ID: 77125155-1

*Phlegmariurus subtrifoliatus* (Brownlie) A.R.Field & Bostock **comb. nov.** Basionym: *Lycopodium subtrifoliatum* Brownlie, Nova Hedwigia Beih., 55 (Pterid. Fl. Fiji) 24. 1977. Synonyms: *Huperzia subtrifoliata* (Brownlie) Holub Folia Geobot. Phytotax. 26(1): 93. 1991. Type: Fiji. *G.Brownlie 1287* (holo: CHR! [CHR341227]).

*Phlegmariurus talamauanus* (Alderw.) A.R.Field & Bostock **comb. nov.** Basionym: *Lycopodium talamauanum* Alderw., Bull. Jard. Bot. Buitenzorg, ser. 2,28:45. 1918. Synonym: *Huperzia talamauana* (Alderw.) Holub, Geobot. Phytotax. 20(1): 77. 1985. Type: Sumatra [Indonesia]. *Bünnemeijer 814* (holo: BO, iso: BONN-Nessel, L! [L57395]). IPNI ID: 77125156-1

*Phlegmariurus terrae-guilelmii* (Herter) A.R.Field & Bostock **comb. nov.** Basionym: *Lycopodium terrae-guilelmii* Herter, Bot. Jahrb. Syst. 54:229. 1916. Synonym: *Huperzia terrae-guilelmii* (Herter) Holub, Geobot. Phytotax. 20(1): 77. 1985. Type: New Guinea. *Ledermann 9602* (holo: B). IPNI ID: 77125157-1

*Phlegmariurus tetrastichus* (Kunze) A.R.Field & Bostock **comb. nov.** Basionym: *Lycopodium tetrastichum* Kunze, Bot. Zeit. (Berlin) 6:99. 1848. Synonym: *Huperzia tetrasticha* (Kunze *ex* Alderw.) Holub, Geobot. Phytotax. 20(1): 77. 1985. Type: Java [Indonesia]. *Zollinger 2460* (iso: G, HBG, L! [L57313]). IPNI ID: 77125159-1

*Phlegmariurus tetrastichoides* (A.R.Field & Bostock) A.R.Field & Bostock **comb. nov.** Basionym: *Huperzia tetrastichoides* A.R.Field & Bostock, Austrobaileya 7(4): 711. 2008. Type: Australia. *A.R.Field & O.Rawlins 1139* (holo: BRI, iso: CNS). IPNI ID: 77125158-1

*Phlegmariurus toppingii* (Herter) A.R.Field & Bostock **comb. nov.** Basionym: *Urostachys toppingii* Herter, Bot Arch. 3:15. 1923. Synonym: *Huperzia toppingii* (Herter) Holub, Geobot. Phytotax. 20(1): 77. 1985. Type: Philippines. *D.L.Topping 1096* (iso: BONN-Nessel, NY! [NY127365], US! [US134342]). IPNI ID: 77125160-1

*Phlegmariurus tournayanus* Lawalrée, Acta Bot. Acad. Sci. Hung. 19:197. 1973. Synonym: *Huperzia tournayana* (Lawalrée) Holub, Geobot. Phytotax. 20(1): 77. 1985. Type: Congo. *J.Louis 4012* (iso: P! [P507738]).

*Phlegmariurus trifoliatus* (Copel.) A.R.Field & Bostock **comb. nov.** Basionym: *Lycopodium trifoliatum* Copel., Bernice P. Bishop Mus. Bull. 59:7. 1929. Synonym: *Huperzia trifoliata* (Copel.) Holub, Geobot. Phytotax. 20(1): 77. 1985. Type: Fiji. *H.E.Parks 20225* (holo: UC! [351031], iso: NY! [NY127366]). IPNI ID: 77125161-1

*Phlegmariurus trigonus* (C.Chr.) A.R.Field & Bostock **comb. nov.** Basionym: *Lycopodium trigonum* C.Chr., Dansk Bot. Ark. 7:191. 1932. Synonym: *Huperzia trigona* (C.Chr.) Tardieu, Adansonia sér. 2, 10: 20. 1970. Type: Madagascar. *H.Perrier 8270* (holo: P! [P466613]). IPNI ID: 77125162-1

*Phlegmariurus ulicifolius* (Sw.) S.R.Ghosh, Pterid. Fl. E. India 64. 2004. Basionym: *Lycopodium ulcifolium* Sw., Syn. Fil. 177. 1806. Synonym: *Huperzia ulcifolia* (Sw.) Trevis., Atti Soc. Ital. Sci. Nat. 17: 248. 1874. Type: India orientalis.

*Phlegmariurus varius* (R.Br.) A.R.Field & Bostock **comb. nov.** Basionym: *Lycopodium varium* R.Br., Prodr. 165. 1810. Synonym: *Huperzia varia* (R.Br.) Trevis., Atti Soc. Ital. Sci. Nat. 17:247. 1874. Type: Australia. *R.Brown Iter Austral. 124* (isolecto: BM!, K!). Heterotypic synonyms: *Lycopodium billardierei* Spring, Bull. Acad. Roy. Sci. Belg. 8:516 1841; *Huperzia billardierei* (Spring) Rothm., Feddes Repert. Spec. Nov. Regni Veg. 54(1) 1944. Type: Nova Hollandia [Australia] *La Billardière s.n*. (syn: P-Jussieu!). *Lycopodium novae-zelandicum* Colenso, Trans. & Proc. New Zealand Inst. 19: 275. 1887; *Huperzia novae-zelandica* (Colenso) Holub, Folia Geobot. Phytotax. 20:75 (1985); Type: New Zealand (holo: WELT). *Lycopodium flagellaria* Bory in Duperry, Voy. monde, Bot. 1: 248 t. 26. 1829. Type: Novelle Irlande [Papua New Guinea] *Durville & Lesson s.n*. (syn: ?); *d’Offack s.n*. (syn: P). *Lycopodium pachystachyum* Desv. *ex* Poir., Encycl. suppl. 3: 544. 1813 [1814]. Type: Novelle Hollande [Australia]. *s.c. s.n*. (holo: P). IPNI ID: 77125163-1

*Phlegmariurus whitfordii* (Herter) A.R.Field & Bostock **comb. nov.** Basionym: *Urostachys whitfordii* Herter, Bot. Arch. 3:14. 1923. Synonym: *Huperzia whitfordii* (Herter) Holub, Geobot. Phytotax. 20(1): 78. 1985. Type: Philippines. *H.N.Whitford 798* (holo: PNH lost 1945, iso: MICH! [MICH1191097], US! [US134345]). Heterotypic synonyms: *Lycopodium magnusianum* Herter, Hedwigia 49:91. 1909. *Huperzia magnusiana* (Herter) Holub, Geobot. Phytotax. 20(1): 74. 1985. Type: Philippines. *M.S.Clemens* (holo: B!). IPNI ID: 77125164-1

*Phlegmariurus xiphophyllus* (Baker) A.R.Field & Bostock **comb. nov.** Basionym: *Lycopodium xiphophyllum* Baker, Handb. fern-allies 12.1887. Synonym: *Huperzia xiphophylla* (Baker) Tardieu, Adansonia sér. 2, 10: 20. 1970. Type: Madagascar. *R.Baron 4512* (holo: K! [K351213]). IPNI ID: 77125165-1

*Phlegmariurus yandongensis* Ching & C.F.Zhang,Bull. Bot. Res., Harbin3(3): 2. 1983. Type: China. *K.Y.Shing 256*.

*Phlegmariurus yunnanensis* Ching, Acta Bot. Yunnan. 4(2): 121. 1982. Type: China. *K.M.Feng 7274* (holo: PE).
